# An Experimental Investigation on the Foliation Strike-Angle Effect of Layered Hard Rock under Engineering Triaxial Stress Path

**DOI:** 10.3390/ma16175987

**Published:** 2023-08-31

**Authors:** Zhaofeng Wang, Guangliang Feng, Xufeng Liu, Yangyi Zhou

**Affiliations:** 1 State Key Laboratory of Geomechanics and Geotechnical Engineering, Institute of Rock and Soil Mechanics, Chinese Academy of Sciences, Wuhan 430071, China; zfwang@whrsm.ac.cn (Z.W.); lxfbym@163.com (X.L.); 2 University of Chinese Academy of Sciences, Beijing 100049, China; 3 Key Laboratory of Ministry of Education on Safe Mining of Deep Metal Mines, Northeastern University, Shenyang 110819, China; zhouyangyi@mail.neu.edu.cn

**Keywords:** layered rock, strike angle, foliation, true triaxial stress, anisotropy

## Abstract

Deep underground engineering encounters substantial layered hard rock formations, and the engineering triaxial stress path involves an increase in maximum principal stress, constant intermediate principal stress, and a decrease in minimum principal stress. However, previous research has focused on rock layer angles under conventional triaxial stress conditions, disregarding the influence of foliation strike angles in engineering triaxial stress scenarios. This study experimentally investigates the effects of foliation strike angles on layered hard rock under an engineering triaxial stress path. To account for the brittleness of layered hard rock, we propose a specific small sample-processing method tailored to the foliation strike angle. True triaxial loading tests are conducted on steep, thin slate samples with two different loading orientations, accompanied by acoustic emission monitoring. Results indicate that the strength under a traditional true triaxial compression condition is similar for specimens with 90° and 0° strike angles. Stress–strain curves show that larger deformations occur perpendicular to bedding planes, while surface fractures propagate exclusively along the bedding planes. Mechanical responses differ significantly between specimens subjected to the engineering triaxial stress path at 0° and 90° strike angles compared to conventional true triaxial loading tests, with a lower bearing capacity and differentiated intermediate and minimum principal strains in the 0° case. Conversely, the 90° case exhibits a higher bearing capacity, consistent deformation, and more acoustic emission events. Numerical simulations comparing plastic zone sizes during actual underground excavation support these conclusions. These findings highlight the effects of foliation strike angles, favoring the 90° strike-angle configuration for excavation activities and providing enhanced stability in the surrounding rock mass.

## 1. Introduction

Excavation activities in underground engineering disturb the stress equilibrium of the rock mass and induce the redistribution of the original stress [1]. As a consequence, the rocks within the stress concentration zone may surpass their bearing capacity, leading to a cascade of engineering disasters, including spalling, collapse, and, in severe scenarios, rock bursts [2]. On the other hand, underground engineering encompasses a substantial number of stratified rock formations [3]. Under varying stress conditions, the anisotropy inherent in these layered rock formations profoundly influences the direction, scope, and mechanisms of their failure, eventually resulting in plate fractures, tilting deformations, and other catastrophic events [4] that gravely jeopardize construction safety. In the context of deep underground engineering involving layered rock formations, the meticulous choice of the excavation direction plays a pivotal role in disaster prevention [5]. Hence, it becomes crucial to investigate the anisotropy arising from the initial foliation of these layered rock formations under engineering stress paths, especially in the context of deep underground projects.

The cornerstone of experimental research lies in comprehending the actual stress paths that layered rock masses encounter in engineering sites. In situ rock experiences a non-uniform three-dimensional stress state, particularly under deep burial conditions with elevated stress levels, leading to significant loading and unloading of the surrounding rock during tunnel excavation [6,7,8,9,10,11]. As a result, the stress-path effect emerges as a prominent characteristic of deep rock engineering. However, measuring the disturbed stress path during rock excavation presents challenges, and the reliability of the data cannot be easily validated [12]. Due to the stress path’s dependence on factors like the original stress, excavation method, and spatial location of the tunnel, there is no need to consider all potential paths in laboratory tests. During tunnel excavation, the tangential stress at the position perpendicular to the maximum principal stress (σ_1_) experiences a sharp increase, while the radial stress (σ_3_), i.e., the minimum principal stress, diminishes to a lesser value. Concurrently, the axial stress (σ_2_), i.e., the intermediate principal stress, remains relatively constant [13,14,15]. The stress concentration position is where the surrounding rock undergoes significant damage and becomes the primary focus of engineering hazard prevention and control.

Laboratory tests are extensively employed to investigate the deformation and failure behavior of rocks under realistic engineering conditions. Numerous experimental studies have been conducted to explore the rock’s response in such settings, with a predominant focus on both conventional triaxial and true triaxial conditions [16]. In conventional triaxial tests, the assumption is often made that the intermediate principal stress is equal to the minimum principal stress, while true triaxial conditions consider these stresses to be unequal [16]. Both types of tests primarily concentrate on scenarios involving continuous monotonic loading. It is important to note that in actual underground engineering scenarios, rocks experience a complex stress path that involves both loading and unloading [17]. Regrettably, these conventional tests have limitations in adequately reflecting the stress-path adjustment process of the surrounding rock during excavation. To address this issue, a series of loading and unloading tests were conducted to investigate the stress-path effect on rock mechanical properties. These experimental investigations encompassed both conventional triaxial stress conditions [14,18] and true triaxial stress conditions [19,20]. Nevertheless, it is noteworthy that the majority of these experimental studies have primarily focused on nearly isotropic rocks, leaving a significant gap in research concerning the deformation and failure behavior of layered rock formations under engineering stress paths [17,21].

The unique foliation structure of layered rock formations presents inherent challenges in conducting experimental research that accounts for both the actual engineering stress path and the influence of foliation. Nevertheless, significant attention has been devoted to investigating the mechanical properties of these rocks during loading and unloading [18]. Previous studies have highlighted that the deformation and failure behavior of layered rocks under loading and unloading conditions are noticeably influenced by the orientation of foliation [22,23,24]. However, a scarcity of research persists concerning the specific loading–unloading stress-path effect on layered rocks, particularly under true triaxial stress conditions. The lack of true triaxial testing conditions has led to a predominant focus on the dip angle’s influence in conventional triaxial studies concerning the anisotropy of layered rock formations. These studies specifically explore the foliation angle within the maximum and minimum principal stress planes [22,23,24]. However, what has been overlooked in these investigations is the influence of the foliation strike angle in space, which pertains to the angle within the intermediate and minimum principal stress planes. Consequently, a comprehensive understanding of the deformation and failure characteristics displayed by layered rocks with varying foliation strike angles under engineering triaxial stress paths is still to be attained.

Several constitutive models for layered rock masses have been established through indoor experimental research [25]. These mechanical models can be broadly classified into two types: independent models and equivalent models, distinguished by how they handle bedding planes. Independent models isolate the rock mass from the bedding planes and can be further categorized as explicit or implicit methods based on their representation of bedding planes [26]. Explicit methods involve separate modeling of bedding planes, as demonstrated by the jointed rock mechanics model within the discrete element method, which defines distinct mechanical models for rock layers and bedding planes [27]. In contrast, implicit methods combine the rock matrix with the bedding planes to form a composite body, directly considering criteria and parameters for both the matrix and bedding planes in the mechanical model [28]. Equivalent models, on the other hand, treat the entire rock mass as a homogenous transversely isotropic body with properties equivalent to bedding planes [29,30]. Moreover, in recent years, a series of data-driven models for layered rock formations have emerged, employing new techniques like machine learning [31,32]. Despite the availability of numerous models, there remains a significant scarcity of suitable models that can accurately address the deformation and failure of layered rock formations in deep engineering projects. This limitation arises from a limited understanding of how the foliation strike angle influences layered hard rock under engineering triaxial stress conditions.

This study conducted a comprehensive experimental investigation to reveal the effects of the foliation strike angle on layered hard rock under engineering triaxial stress paths. Section 2 presents a detailed introduction to the characteristics and sampling information of the rock materials utilized in the study, along with the experimental equipment and testing procedures. Additionally, a specific small-sample processing method tailored to the brittle nature of the layered hard rock, considering the foliation strike angle of the rock mass, is described. Section 3 presents the final experimental results, with Section 3.1 focusing on the outcomes of conventional true triaxial tests, while Section 3.2 provides a comprehensive analysis of the effects of the foliation strike angle on layered hard rock under engineering triaxial stress paths. This analysis covers various aspects, including deformation, acoustic emission characteristics, strength, and failure characteristics. In Section 4, the discussion section, a comparison is made between the deformation and failure characteristics of layered rock formations under conventional true triaxial compression paths and engineering triaxial stress paths. This comparison emphasizes the necessity of conducting engineering triaxial stress path tests, as highlighted in Section 4.1. Moreover, numerical simulation is employed to verify the effects of the foliation strike angle on layered hard rock under engineering triaxial stress paths in a practical engineering scenario in Section 4.2. The findings from the numerical simulation provide valuable engineering insights for the excavation of deep-layered rock formations.

## 2. Methodology

### 2.1. Materials

**Figure 1:**
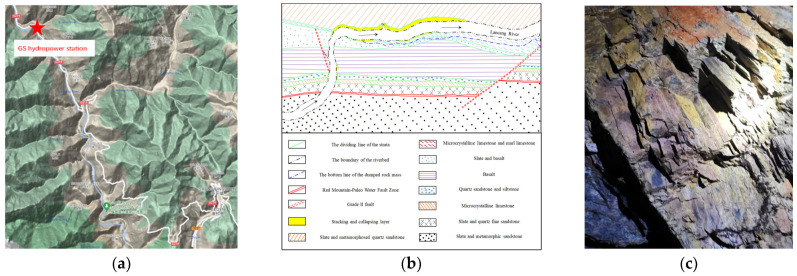
Sampling information: (**a**) Location of the hydropower station; (**b**) Geological map around the hydropower station; (**c**) Slate exposed by the exploration tunnel in the hydropower station.

The rock samples for this study were collected from steep, thin-layered slates in the powerhouse area of a hydropower station situated in Yunnan Province, China. The hydropower station is currently in the feasibility study stage, as illustrated in Figure 1a. The river flows from north to south in the upstream section, entering the dam area, and then changes its course in the middle section, flowing from west to east. Finally, in the downstream section, it flows from north to south, exiting the dam area, as depicted in Figure 1b. The terrain in the upper dam area exhibits relatively poor integrity, with longitudinal valleys in the upstream and downstream sections, as well as a transverse valley in the middle section. The strata in the area comprise slate, limestone, mudstone, and basalt, as well as slate and sandstone. The overall strike of the rock layers is northwest, and they steeply dip in the upstream section. In addition, loose layers are widely distributed throughout the region. These slates, shown in Figure 1c, are prone to experiencing fracture phenomena along their foliation. The primary objective of this study is to analyze the behavior of the thin slate under true triaxial loading and unloading conditions and to assess the stability of the surrounding rock during tunnel excavation.

**Figure 2:**
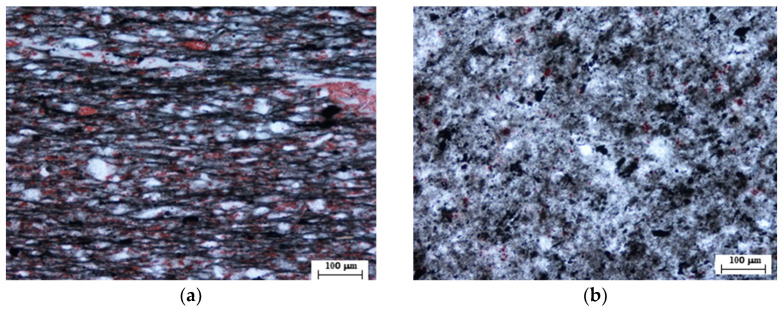
Polarized microscopic image of the slate: (**a**) Perpendicular to foliation direction; (**b**) Parallel to foliation direction.

Several thin-slate blocks were extracted from the hydropower station and subjected to thin-section observations under cross-polarized light microscope for mineral composition and structure identification (Figure 2). As shown in Figure 2a, the mineral structure of the slate exhibits distinct directional distribution characteristics perpendicular to its foliation. Based on identification results, the slate’s mineral content primarily comprises argillaceous (~40–45%), carbonate (~20–25%), felsic (~20–25%), muscovite (~1–2%), and opaque mineral (~1–2%). The argillaceous component is mostly composed of clay minerals, with particle sizes of most minerals being less than 0.005 mm. The calcareous content is mostly allomorphic microcrystalline, with mineral particles mostly less than 0.05 mm, scattered among felsic clastic particles. The felsic clasts are generally angular, and mineral particle sizes are mostly less than 0.06 mm, mainly composed of well-sorted quartz that is roughly oriented along the long axis. Muscovite is mostly in strip shape with diameters less than 0.1 mm, and it is predominantly directionally distributed along the long axis. Opaque minerals mostly consist of carbon and iron in small amounts. The slate is classified as a silty slate under the microscope, and it is generally characterized by residual silty argillaceous texture and plate structure.

### 2.2. Preparations for Fragile-Layered Hard Rock Sample

The intrinsic foliation structure of slate can lead to variations in its mineral composition and structure, resulting in significant fluctuations in test results and reducing the repeatability of experimental data. To address this issue and ensure specimen homogeneity, all specimens were derived from a single rock block, and those with visible defects were discarded. Due to the inherent brittleness of hard rocks, layered hard rocks present even greater challenges in processing [33]. Hence, this study proposes a specific small-sample processing method tailored to the foliation strike angle.

Utilizing small samples offers the advantage of easier processing compared to larger ones. Moreover, following the ISRM Suggested Method [16] for rock true triaxial compression tests in Section 4.2, maintaining the specimen length parallel to the major principal stress twice the width can avoid end effects and prevent the sample size from influencing the stress–strain behavior and failure mode of the rock samples. Notably, various authoritative experts have employed smaller dimensions, such as 15 mm × 15 mm × 30 mm [34], 19 mm × 19 mm × 38 mm [35], and 35 mm × 35 mm × 70 mm [36], for rock true triaxial compression tests, and their research findings on the effects of intermediate principal stress and true triaxial strength criteria have gained widespread recognition.

**Figure 3:**
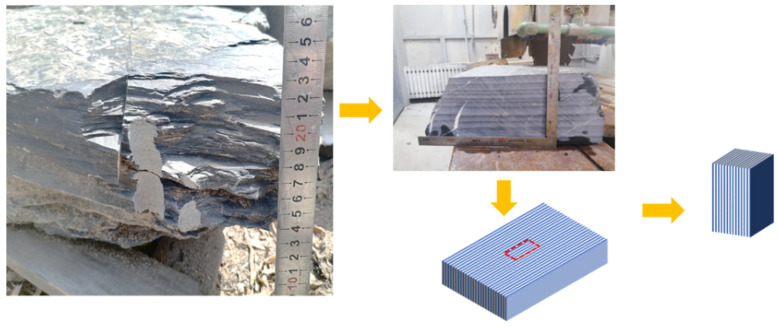
Schematic diagram of a specific small-sample processing method tailored to the foliation strike angle.

**Figure 4:**
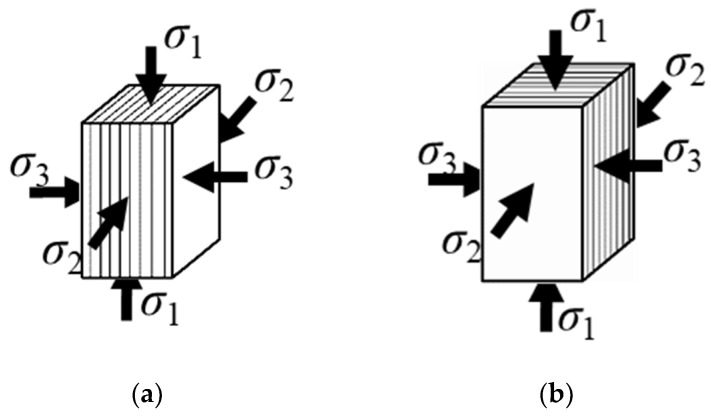
Two types of specimens with different loading orientations of the foliation: (**a**) Case I, β = 0°, ω = 0°; (**b**) Case II, β = 0°, ω = 90°.

In this study, small samples were utilized to address challenges in maintaining integrity during processing. To control different foliation angles within the specimens, a step-by-step rock sample processing method was devised, as illustrated in Figure 3. Initially, a large rock block was cut into 30-mm thick rock plates parallel to the schistosity direction. Then, the outline of the specimen was marked on the rock plate according to the loading angle. Subsequently, the rock plate was cut to create a specimen slightly larger than the standard size. Finally, the specimen was polished to meet the required geometric dimensions and tolerance [16]. The resulting rectangular prismatic specimens had final dimensions of 25 mm × 25 mm × 50 mm, as depicted in Figure 4. Two types of rock samples were prepared: Case I with β = 0°, ω = 0°; and Case II with β = 0°, ω = 90°. Here, β represents the angle between the foliation plane and the maximum principal stress σ_1_ in the σ_1_–σ_3_ plane, while ω represents the angle between the foliation plane and the intermediate principal stress σ_2_ in the σ_2_–σ_3_ plane. These measures were implemented to minimize external factors’ impact and enhance the accuracy and reliability of the experimental results in investigating the fundamental mechanical properties of rocks.

### 2.3. Testing Equipment

This study utilized the fast unloading and tensile test device for rigid true triaxial compression of soft rock at Northeastern University [33]. The device is powered by electricity and employs the “three rigidities” loading mode to effectively mitigate the disadvantage of a large impact on the oil pipeline during unloading in hydraulic power mode. It is capable of conducting true triaxial static loading and unloading tests, as well as instantaneous unloading tests in the σ_3_ direction. The maximum loading capacity of the device is 300 kN in the vertical direction (σ_2_), and 200 kN and 100 kN in the horizontal directions of σ_1_ and σ_3_, respectively. Three grating deformation extensometers (GDE) were utilized to measure the deformations in the ε_1_, ε_2_, and ε_3_ directions (where ε_1_, ε_2_, and ε_3_ correspond to the three strain components of σ_1_, σ_2_, and σ_3_). Additionally, an acoustic emission (AE) monitoring system was installed in the device to allow for real-time AE information monitoring during the test.

### 2.4. Experimental Procedures

**Figure 5:**
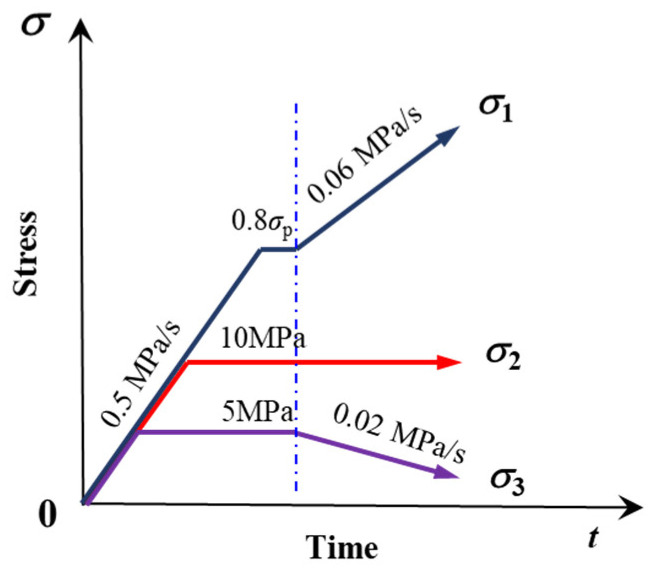
Engineering triaxial stress path adopted in this paper.

The steep occurrence of slate in the hydropower station necessitates the division of loading orientation (β, ω) of foliation in tests into two categories: (0°, 0°) and (0°, 90°), referred to as Case I and II, respectively. Here, β represents the intersection angle between the foliation and σ_1_, and ω is the intersection angle between the strike of the foliation and σ_2_ direction, as illustrated in Figure 4. Building on the foregoing section, we employ a typical loading–unloading stress path model where σ_3_ declines, σ_1_ increases, and σ_2_ remains constant. In this study, two types of tests are conducted: true triaxial loading tests and true triaxial loading and unloading tests. The former serves as a reference for setting stress levels for the latter. Specifically, the stress level in true triaxial loading tests is set at σ_3_ = 5 MPa and σ_2_ = 10 MPa, and three sets of tests are conducted at each loading angle. In true triaxial loading and unloading tests, the initial stress levels are established as σ_3_ = 5 MPa, σ_2_ = 10 MPa, and σ_1_ = 0.8 σ_p_, where σ_p_ is the average peak strength of the specimen in the loading test. During true triaxial loading and unloading tests, the stress path proceeds as follows: First, σ_1_, σ_2_, and σ_3_ are simultaneously applied at a rate of 0.5 MPa/s to 5 MPa; then, σ_3_ is maintained constant, and σ_1_ and σ_2_ are simultaneously applied at a rate of 0.5 MPa/s to 10 MPa; next, σ_2_ is held constant, and σ_1_ is applied at a rate of 0.5 MPa/s to 0.8 σ_p_ and sustained for 30 s. Finally, σ_1_ is applied at a rate of 0.06 MPa/s, and σ_3_ is unloaded at a rate of 0.02 MPa/s until the specimen fails completely. The stress path is depicted in detail in Figure 5, and the specific test scheme is outlined in Table 1, with three sets of each loading orientation tested. During the test, we monitor Acoustic Emission (AE) information using a PCI-2 system produced by PAC. Four Nano-30 AE sensors, with a center frequency of 200–750 kHz, are fastened on the loading block close to the specimen with a wide rubber band. The threshold of the AE signal is set to 30 dB, and the sampling frequency is set to 1 MHz.

## 3. Results and Analysis

### 3.1. Traditional True Triaxial Compression Tests

**Figure 6:**
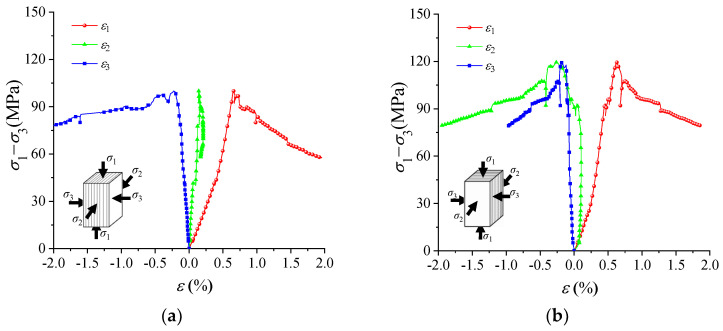
Typical stress–strain curves of specimens with two loading orientations: (**a**) Case I; (**b**) Case II.

**Figure 7:**
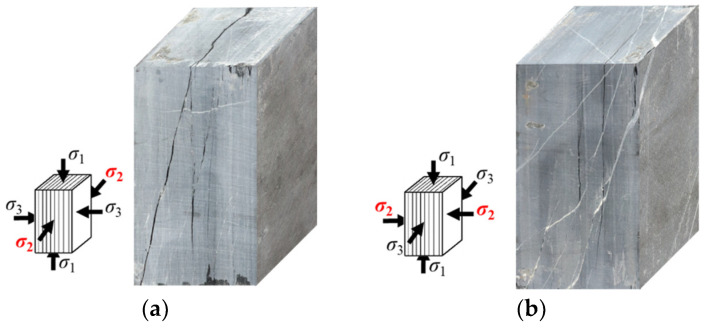
Typical failure modes of specimens with two loading orientations: (**a**) Case I; (**b**) Case II.

Table 2 presents the true triaxial compression strength of specimens with two loading orientations. The strength of the specimen with ω = 90° is slightly higher than that with ω = 0°, which is consistent with the true triaxial compression results reported for schist, gneiss, and metamorphic siltstone [33]. When ω = 90°, σ_2_ is perpendicular to the strike of foliation, resulting in a relatively large normal stress on foliation that inhibits foliation cracking and leads to higher strength. The difference in strength between the specimens with two loading orientations is small due to the relatively low differential stress (σ_2_–σ_3_), which weakens the strengthening effect caused by normal stress. For deformation, the normal deformation characteristics of foliation exhibit little difference under the two loading orientations when ω = 90°, as illustrated in Figure 6. In terms of failure, cracking along foliation occurs similarly under the two loading orientations, as shown in Figure 7.

### 3.2. Engineering Triaxial Stress Path Tests

#### 3.2.1. Deformation and AE Characteristics

**Figure 8:**
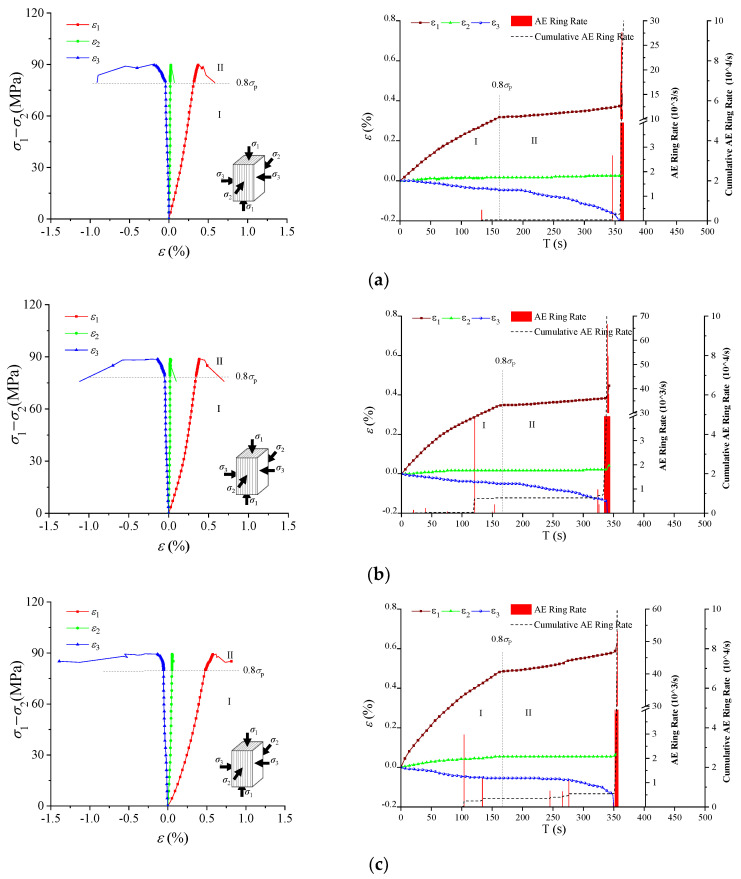

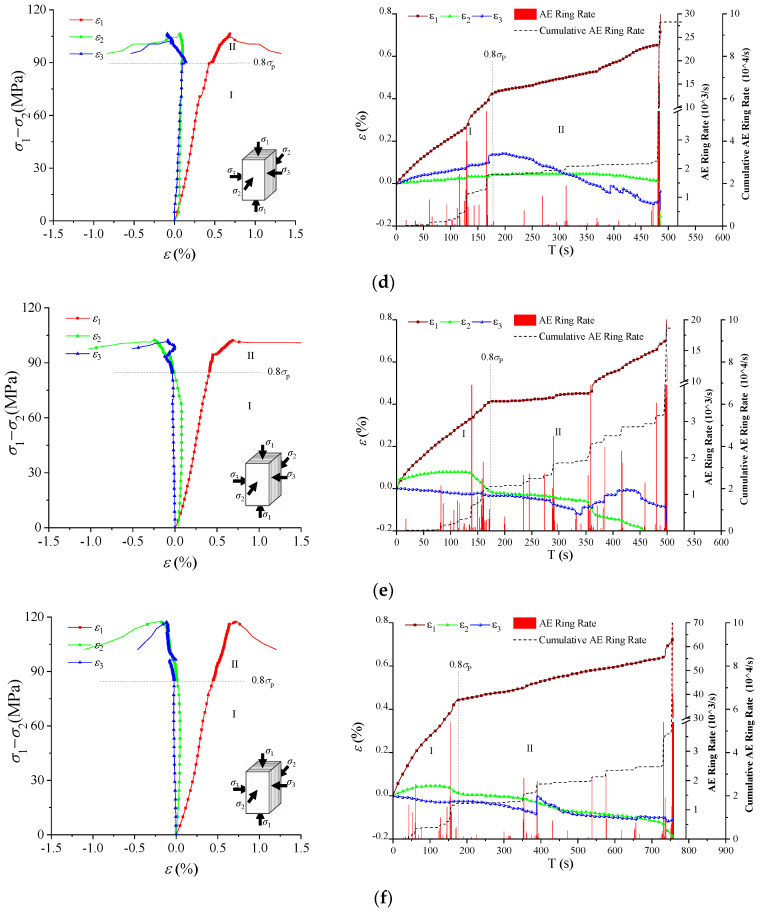
Full stress-strain curve and acoustic emission (AE) evolution characteristics of the specimens in Case I and II under true triaxial loading and unloading: (**a**) Case I, Specimen 1; (**b**) Case I, Specimen 2; (**c**) Case I, Specimen 3; (**d**) Case II, Specimen 1; (**e**) Case II, Specimen 2; (**f**) Case II, Specimen 3.

Figure 8 presents stress–strain curves and AE evolution data for three sets of specimens with two loading orientations during loading and unloading. In Case I, the maximum and minimum principal strains (ε_1_ and ε_3_, respectively) change significantly during the σ_1_ loading stage (Stage I), while the intermediate principal strain (ε_2_) remains relatively constant, consistent with the characteristics of true triaxial compression testing (Figure 8a–c). During loading and unloading (Stage II), the strain changes in all directions are similar to those in Stage I, where ε_3_ increases nonlinearly, demonstrating a strong sensitivity to loading and unloading. Case I’s strain variation characteristics in Stage II have significant differentiation: compression deformation in the σ_1_ direction is noticeable, deformation in the σ_2_ direction is approximately constant, and the expansion deformation in the σ_3_ direction is evident. This expansion deformation increases nonlinearly as the stress approaches the specimen’s bearing capacity. These deformation differences are related to the foliation structure and stress conditions. In this loading orientation, σ_2_ is parallel to the foliation, and σ_3_ is parallel to the normal direction of foliation. At Stage II, the normal deformation of foliation is mainly due to elastic deformation and microcrack development, with the former primarily caused by the Poisson effect induced by σ_1_ loading and the rebound deformation caused by σ_3_ unloading, and the latter primarily due to the combined effect of stress induction and foliation structure, leading to the preferential formation of microcracks on foliation. The approximate invariance of ε_2_ is due to the small Poisson ratio in the direction of parallel foliation and the unchanged σ_2_. Although ε_3_ has a significant increase process in Stage II for Case I, its AE signal is not evident, and the AE impact rate increases significantly only when failure is imminent, indicating that the failure process is more abrupt when the normal direction of foliation is unconstrained.

Compared to Case I, the deformation and AE characteristics of Case II differ significantly, as demonstrated in Figure 8d–f. Firstly, there is no significant deformation differentiation. At Stage II, ε_2_ and ε_3_ are small, and local abrupt changes occur. Secondly, the AE signals are distributed uniformly throughout Stage II. This is mainly due to the constraint effect of σ_2_ on the foliation, which restricts the normal deformation of the foliation to some extent and enhances lateral deformation uniformity. However, the slate has a strong anisotropy, making it susceptible to cracking along the foliation. Even with the constraint of σ_2_, the local foliation may crack under compression-induced tensile strain at high stress levels. Due to the large normal stress on the foliation, the local micro-crack of the foliation results in a strong energy release, local deformation mutation, and a sudden increase in the AE impact rate. Additionally, the peak strain of Case II specimens is higher, suggesting a slow microcrack evolution process and a more gradual failure process.

#### 3.2.2. Strength and Failure Characteristics

Table 3 presents the stress levels of specimens with two loading orientations at the point of final failure. In Case I, each specimen experiences an σ_3_ greater than zero, indicating that failure occurs during Stage II. In contrast, in Case II, failure does not occur during the σ_3_ unloading stage, and the final failure happens during the σ_1_ loading stage. The findings suggest that specimens in Case I are more sensitive than those in Case II, and the latter demonstrate a stronger ability to withstand stress adjustment induced by excavation activities, thus exhibiting greater stability.

**Figure 9:**
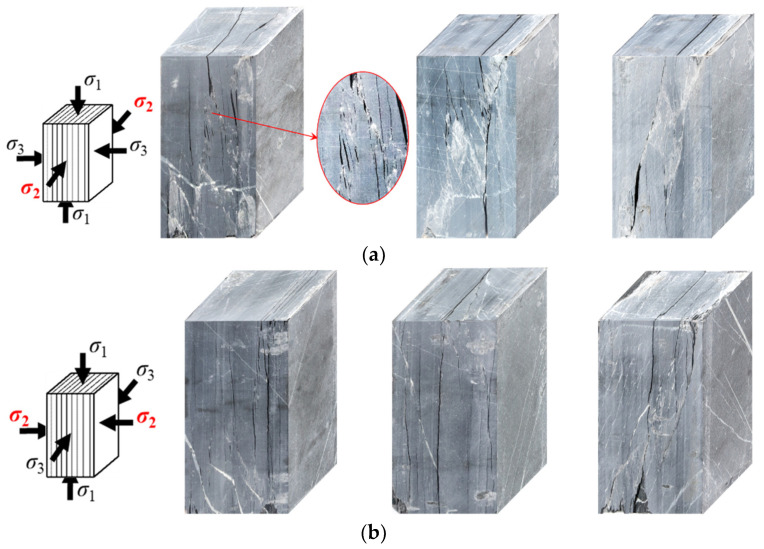
Fracture mode of the specimens with two loading orientations: (**a**) Case I, (**b**) Case II.

Figure 9 depicts the fracture patterns of the specimens in two loading orientations. In general, cracking in both cases occurs along the foliation. In Case I, the specimens mainly experience tensile-shear failure, with numerous tensile micro-cracks observed along the shear path and occasional kinking of the foliation. Meanwhile, Case II specimens only experience tensile failure along the foliation. This fracture behavior is consistent with the deformation and AE characteristics observed earlier. In Case I, foliation is unconstrained by σ_2_, resulting in significant normal expansion at Stage II, which then culminates in specimen collapse along the weak foliation plane accompanied by strong energy release. In contrast, although σ_2_ constraints the foliation in Case II, it is insufficient to prevent micro-crack initiation on the highly anisotropic foliation. AE results suggest that the fracture process is gradual, ultimately leading to tensile fracture along the foliation. Notably, there is no tensile-shear failure collapse, as seen in Case I. This could be attributed to the increase in normal stress on the potential shear plane by σ_2_, rendering shear slip movement difficult to trigger.

## 4. Discussions

### 4.1. Comparisons between Traditional and Engineering True Triaxial Stress Paths

Presently, the majority of investigations pertaining to the foliation strike angle effect in layered hard rock adopt conventional true triaxial compression paths [16,33]. However, it is essential to recognize that real engineering stress paths display distinctive characteristics. Specifically, during tunnel excavation, the tangential stress perpendicular to the maximum principal stress (σ_1_) undergoes a marked increase, while the radial stress (σ_3_), corresponding to the minimum principal stress, diminishes to a lesser extent. However, the axial stress (σ_2_), representing the intermediate principal stress, remains relatively constant [13,14,15]. In this study, we term this particular stress path the “engineering true triaxial stress path.” Remarkably, the foliation strike angle effect exhibits noteworthy differences between these two stress paths. To comprehensively assess these distinctions, this research introduces two quantitative evaluation indices: strength and deformation difference ratio (*DDR*). The *DDR* index is computed through the following method:

(1) $$DDR=\frac{\left|\varepsilon_{2}^{peak}\right|}{\left|\varepsilon_{3}^{peak}\right|}\;\;$$

where the $\varepsilon_{2}^{peak}$ and $\varepsilon_{3}^{peak}$ denote the intermediate and minimum principal strains, respectively, occurring under peak stress conditions.

Evidently, the strength index effectively characterizes the bearing capacity of the layered rock mass influenced by the foliation strike angle effect, while the DDR index quantifies the deformation discrepancies in two lateral directions. Figure 4 illustrates Case I, where the foliation planes align parallel to the intermediate principal strain direction, and Case II, where the foliation planes align parallel to the minimum principal strain direction. The ratio of intermediate-to-minimum principal strains (DDR) at the peak moment reflects the deformation disparities between the foliation plane direction and the direction perpendicular to it.

**Figure 10:**
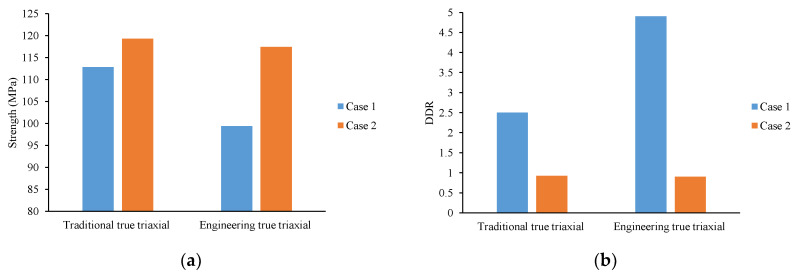
Comparison results between traditional and engineering true triaxial stress path: (**a**) Strength; (**b**) DDR.

In Figure 10, the comparison results between traditional and engineering true triaxial stress paths are presented. For Case I’s strength (see Figure 10a), the engineering true triaxial stress path exhibits a decrease of over 20 MPa compared to the traditional true triaxial stress path. For Case II, the engineering true triaxial stress path decreases by approximately 3 MPa, which is not significantly different from Case I. In terms of DDR for Case I (see Figure 10b), the engineering true triaxial stress path increases by nearly 2.5 in comparison to the traditional true triaxial stress path, while for Case II, the strength of the engineering true triaxial stress path is roughly similar to the traditional true triaxial stress path.

These results challenge the existing research on layered rock masses based on conventional true triaxial paths and emphasize the research value of the engineering path adopted in this study. Regardless of Case I or Case II, the strength and deformation differences observed under the engineering stress path are more pronounced than those under the conventional true triaxial stress path. Conducting experiments based on the conventional true triaxial stress path to reveal mechanisms and establish strength and deformation models may lead to reduced strength values and smaller deformation differences compared to actual underground engineering construction. Consequently, this could result in biased estimates of the surrounding rock bearing capacity and hinder the accurate prediction of deformation in the foliation plane direction and the direction perpendicular to it.

### 4.2. Numerical Verifications and Engineering Insights

The test results presented in Section 3 reveal significant differences in the failure characteristics of the specimens in the two cases. In Case I, the specimen’s ultimate failure mode is complex and exhibits multi-scale tension-shear failure. Initially, the acoustic emission (AE) signal is insignificant, suggesting that the crack along the foliation is either not apparent or consists of micro-fractures, which the AE equipment cannot detect. As σ_3_ is unloaded to a lower value, the outer edge of the specimen’s foliation cracks transfers stress, resulting in the actual stress exceeding the nominal stress. This leads to tensile-shear failure following the weak foliation rapidly, causing a loss of bearing capacity as the foliation is not constrained by σ_2_, and σ_3_ continues to unload. However, in Case II, the tensile failure along the foliation does not lead to a complete loss of bearing capacity, and independent parallel foliations can still bear the load when connected in parallel. The slow-crack evolution process results in significant deformation when reaching peak stress. It is noteworthy that the σ_2_ level in this study is relatively low, and the slate exhibits strong anisotropy. Higher σ_2_ levels may induce a more prominent stress-induced effect, leading to tensile-shear failure along the foliation.

**Figure 11:**
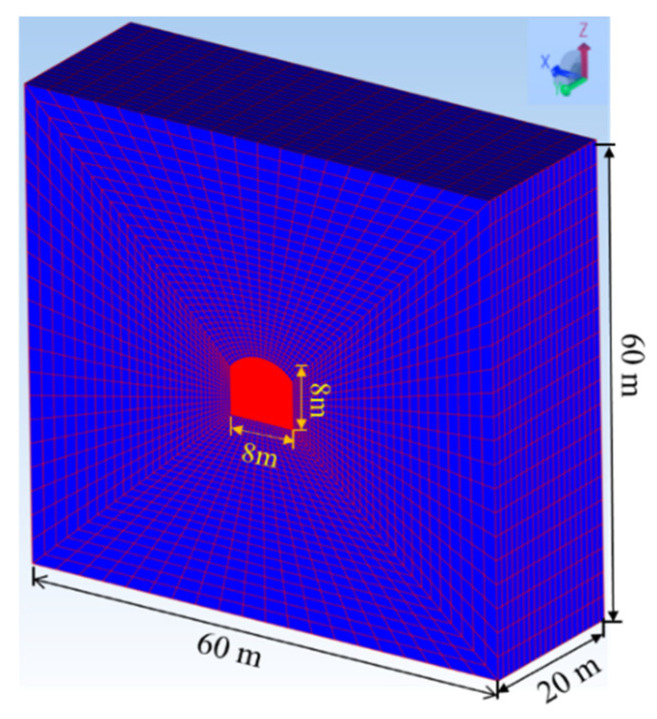
Numerical model applied in this paper.

**Figure 12:**
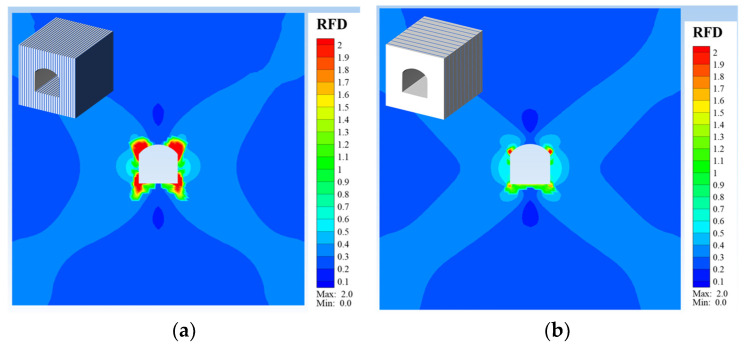
Simulated results: (**a**) The tunnel axis is parallel to the foliation direction, corresponding to Case I; (**b**) The tunnel axis is perpendicular to the foliation direction, corresponding to Case II.

To validate the reasonableness of these test findings, corresponding numerical simulations were conducted. In this section, the Cellular Automata Software for Engineering Rock-mass Fracturing Process (CASRock v1.0), incorporating a mixed anisotropic layered rock mechanics model [30], was utilized for the numerical study. An initial grid model of the existing engineering experimental tunnel was established, as depicted in Figure 11. The numerical model dimensions are 60 m × 60 m × 20 m, with a tunnel shape of a straight-wall circular arch and section dimensions of 8 m × 8 m. The model comprises 56,000 elements. Specific stresses were applied to the model boundaries, and normal displacement constraints were used. The stress values applied to the tunnel model boundaries, based on in situ stress conditions, are shown in Table 4, and the rock mass parameters for the tunnel model, based on geological exploration reports and on-site measurements, are shown in Table 5. Figure 12 illustrates the final simulation results, where Figure 12a corresponds to when “the tunnel axis is parallel to the foliation direction, corresponding to Case I,” and Figure 12b corresponds to when “the tunnel axis is perpendicular to the foliation direction, corresponding to Case II.” In this study, the Rock Fracturing Degree index was employed to evaluate the extent of rock fracturing, with its definition referenced from the previous literature [30,37]. From Figure 12, it is evident that the final failure modes for Case I and Case II are generally similar (i.e., failure concentrates at the left and right arch shoulders and the left and right arch feet). However, the extent of failure and the range of fracturing are much greater in Case I than in Case II. This confirms the conclusions mentioned in Section 3, which state that Case I exhibits lower strength (Table 3), greater deformation differences (Figure 10), more abrupt acoustic emission signals (Figure 8), and more severe failure (Figure 9) than Case II.

**Figure 13:**
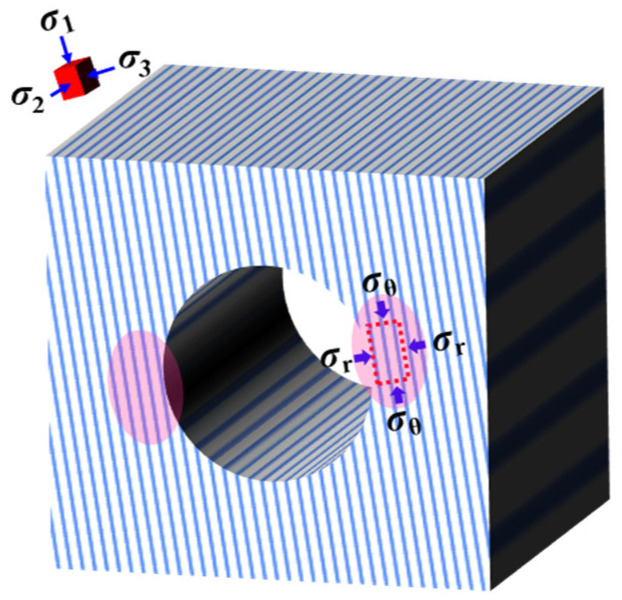
Stress redistribution on cross section after tunnel excavation: σ_θ_ is tangential stress; σ_r_ is radial stress.

Both the test and numerical results provide valuable engineering insights for the excavation of layered rock tunnels. Importantly, it was observed that the stress perpendicular to the direction of maximum principal stress in the tunnel section exhibits significant differentiation. When the tunnel axis intersects with the foliation strike at a small angle, the loading orientation is similar to Case I (Figure 13), and cracking along the foliation occurs due to the low-bearing capacity and limited allowable deformation. In such areas, the close monitoring of deformation development and appropriate support measures are necessary to prevent rock block ejection, falling, or collapse in the shallow surrounding rock. Conversely, when the tunnel axis intersects with the foliation strike at a large angle, the loading orientation is similar to Case II, indicating a high-bearing capacity and substantial allowable deformation, which is more favorable for excavation and provides stronger surrounding rock stability. It is crucial to acknowledge that this study is preliminary, and several factors, such as initial stress level, loading and unloading rate, and foliation dip angle, may influence the mechanical properties of the slate under loading and unloading. However, considering these factors would significantly increase the test workload and cost, maintaining slate homogeneity in large-scale tests would be challenging. To address this, the mechanical model and parameters of the slate will be calibrated based on the laboratory test results, and numerical simulations will be conducted to investigate these factors.

## 5. Conclusions

This study aimed to investigate the influence of the foliation strike angle on deformation and failure in underground engineering excavations. It conducted an experimental investigation on the foliation strike angle effect of layered hard rock under an engineering triaxial stress path. The study employed a specific small-sample processing method tailored to the foliation strike angle to control different foliation angles within the specimens. It analyzed the deformation, failure, acoustic emission, and strength characteristics under the engineering triaxial stress path, successfully overcoming challenges in processing square samples with arbitrary foliation directions and conducting tests under engineering true triaxial stress conditions. Comparisons with traditional true triaxial stress paths were made, and numerical simulations were employed to validate the discovered mechanisms, offering valuable engineering insights. The main conclusions drawn from the study are listed as follows:

1. A novel specific small-sample processing method was proposed, designed to accommodate the foliation strike angle. This method minimized rock sample sizes following the ISRM-suggested method to prevent fracturing during processing and testing. By employing a step-by-step rock-sample processing approach, different foliation angles were controlled within the specimens.
2. The study unveiled the foliation strike angle effect of layered hard rock under the engineering triaxial stress path. Diverging from conventional research focusing on foliation angle in the σ_1_–σ_3_ plane, the study centered on the foliation strike angle effect within the σ_2_–σ_3_ plane. When the foliation was parallel to σ_2_, the specimen experienced complex multi-scale tension-shear failure, initially with insignificant AE signals indicating potential micro-fractures. Unloading σ_3_ led to cracks along the foliation edge, causing tensile-shear failure due to limited constraint from σ_2_. Conversely, when the foliation was parallel to σ_3_, the specimen exhibited less bearing capacity loss, as independent parallel foliations were still able to bear the load. This failure process evolved gradually, marked by numerous small AE events.
3. Comparisons between traditional and engineering true triaxial stress paths demonstrated that irrespective of the foliation direction being parallel to σ_3_ or σ_2_, the strength and deformation differences observed under the engineering stress path were more pronounced than those under the conventional true triaxial stress path.
4. The numerical simulation results strongly validated the foliation strike angle effect of layered hard rock under the engineering triaxial stress path, confirming the lower strength, greater deformation differences, more abrupt acoustic emission signals, and more severe failure observed when the foliation was parallel to σ_2_.

In conclusion, this study represents a significant advancement over conventional methods that focus on σ_1_–σ_3_ plane angle effects in rock. It successfully overcame challenges related to processing layered hard-rock samples and conducting tests under engineering true triaxial stress conditions. Additionally, it is the first study to investigate the foliation strike angle effect within the σ_2_–σ_3_ plane under engineering triaxial stress. The findings lay the groundwork for future experimental research and relevant techniques. Furthermore, the study emphasizes the importance of considering the actual engineering stress path, as tests based on simplified indoor stress paths may lead to underestimated strength and deformation discrepancies compared to actual underground engineering construction. These research insights open new perspectives for subsequent mechanism analysis and simulation. It is important to note that this study exclusively examined two scenarios for one type of layered rock: when the foliation is parallel to σ_3_ or σ_2_. While conventional wisdom suggests that foliation parallel to σ_2_ leads to more severe failure than when it is parallel to σ_3_, our investigation yields quantitative comparison results that uncover potentially greater disparities in deformation and failure between engineering true triaxial and simplified stress paths, surpassing previous assumptions. This research offers valuable guidance for selecting tunnel excavation directions and analyzing engineering failure locations and degrees in layered rock masses for underground engineering construction. Future studies will further explore additional foliation strike angles and more rock types to fully reveal the foliation strike angle effect of layered hard rock under engineering triaxial stress.

## Figures and Tables

**Table 1:** Test scheme for specimens considering the loading orientation of foliation and stress regime.

Orientation of Foliation			Test Type	Initial Stress Regime		
Loading Angle	β (°)	ω (°)		σ_3_ (MPa)	σ_2_ (MPa)	σ_1_ (MPa)
Case I	0	0	Loading	5	10	\
	0	0	Loading and unloading	5	10	0.8 σ_p_
Case II	0	90	Loading	5	10	\
	0	90	Loading and unloading	5	10	0.8 σ_p_

**Table 2:** Strength of specimens with two loading orientations under true triaxial compression.

Loading Angle		Strength of Each Specimen			Average Strength σ_p_
β (°)	ω (°)	(MPa)			(MPa)
0	0	102.5	115.1	120.8	112.8
0	90	129.4	124.4	104.2	119.3

**Table 3:** Stress levels of specimens at final failure for each loading orientation.

Loading Angle		No.	Final Stress Level		
β (°)	ω (°)		σ_3_ (MPa)	σ_2_ (MPa)	σ_1_ (MPa)
0	0	1	1.6	10	100.1
		2	2.1	10	98.6
		3	1.8	10	99.6
0	90	1	0	10	111.9
		2	0	10	112.6
		3	0	10	127.7

**Table 4:** Stress level applied in numerical model.

*σ*_xx_ (MPa)	*σ*_yy_ (MPa)	*σ*_zz_ (MPa)	*σ*_xy_ (MPa)	*σ*_yz_ (MPa)	*σ*_xz_ (MPa)
14.59	24.92	21.99	−1.48	2.68	2.32

**Table 5:** Parameter value of numerical model.

***E*_1_** **(GPa)**	***E*_3_** **(GPa)**	***ν*_11_**	***ν*_13_**	***G*_13_** **(GPa)**	***ρ*** **(kg/m^3^)**
11.55	6.27	0.2	0.4	2.38	2500
***c*_0_** **(MPa)**	***φ*_0_** **(°)**	***c*_r_** **(MPa)**	***φ*_r_** **(°)**	***σ*_t_** **(MPa)**	***σ*_r_** **(MPa)**
28.00	21	1.00	26	1.00	0.10

## Data Availability

All data used during the study appear in the submitted article.
